# A Closer Look at Laryngeal Nerves during Thyroid Surgery: A Descriptive Study of 584 Nerves

**DOI:** 10.1155/2012/490390

**Published:** 2012-06-12

**Authors:** P. V. Pradeep, B. Jayashree, Skandha S. Harshita

**Affiliations:** Department of Endocrine Surgery, Narayana Medical College & Superspeciality Hospital, Chinthareddypalem, Nellore 524002, India

## Abstract

Morbidity after thyroidectomy is related to injuries to the parathyroids, recurrent laryngeal (RLN) and external branch of superior laryngeal nerves (EBSLN). Mostly these are due to variations in the surgical anatomy. In this study we analyse the surgical anatomy of the laryngeal nerves in Indian patients undergoing thyroidectomy. *Materials and Methods*. Retrospective study (February 2008 to February 2010). Patients undergoing surgery for benign goitres, T1, T2 thyroid cancers without lymph node involvement were included. Data on EBSLN types, RLN course and its relation to the TZ & LOB were recorded. *Results*. 404 thyroid surgeries (180 total & 224 hemithyroidectomy) were performed. Data related to 584 EBSLN and RLN were included (324 right sided & 260 left sided). EBSLN patterns were Type 1 in 71.4%, Type IIA in 12.3%, and Type IIB in 7.36%. The nerve was not seen in 4.3% cases. RLN had one branch in 69.34%, two branches in 29.11% and three branches in 1.36%. 25% of the RLN was superficial to the inferior thyroid artery, 65% deep to it and 8.2% between the branches. TZ was Grade 1 in 65.2%, Grade II in 25.1% and Grade III in 9.5%. 31.16% of the RLN passes through the LOB. *Conclusions*. A thorough knowledge of the laryngeal nerves and anatomical variations is necessary for safe thyroid surgery.

## 1. Introduction

Thyroid surgery was associated with high mortality rates in the early nineteenth century. The high mortality (20%) was attributed to the lack of meticulous dissection techniques and asepsis [1]. So much so, in the year 1850 the French Academy of Medicine banned thyroid surgery [2]. With the advent of antiseptic techniques and antibiotics the mortality due to sepsis has disappeared. So also, the refinement in surgical techniques, recognition of the presence of parathyroids, RLN, and need to protect the EBSLN resulted in lesser morbidity. Through understanding of the surgical anatomy has been crucial in decreasing the morbidity. Mostly the morbidity is due to technical failure to identify the vital structures and the variations in the surgical anatomy when the gland is pathologically enlarged. Several studies have been published revealing the anatomy of the laryngeal nerves as seen during thyroid surgery [1, 3–5]. Exposure of EBSLN and individual ligation of superior thyroid artery branches in the medial thyroid space was initially stressed to avoid injury to it [1, 6, 7]. Based on the course of the EBSLN; classifications were also put forward by Cernea et al. and Friedman et al. [4, 8]. EBSLN has several branches to pharynx and thyroid gland apart from the clinically important cricothyroidal branch [9]. The RLN is routinely exposed and traced during all thyroid surgeries. It has been realised that the RLN has extralaryngeal branching and this can be damaged if the individual branches are not taken care of by meticulous dissection [10, 11]. However, no such detailed study has been published from Indian subjects. In this study we analyse the surgical anatomy of the laryngeal nerves both RLN and EBSLN and its variations in Indian patients undergoing thyroid surgeries at a specialised endocrine surgical unit.

## 2. Materials and Methods

This descriptive study based on the retrospective data was conducted at a tertiary care centre in South India during the period February 2008 to 2010. Approval of the Institutional Ethics committee was obtained for publishing the study. All patients give informed consent prior to thyroid surgery. Patients who were operated for benign goitres including toxic goitres and early thyroid cancers (T1/T2 N0M0) were included. Patients undergoing surgery for recurrent goitres and advanced thyroid cancers were excluded. The surgical findings are recorded in predesigned “Operation notes” register. These were later entered in the SPSS software 13vs for the analysis.

**Figure 1:**
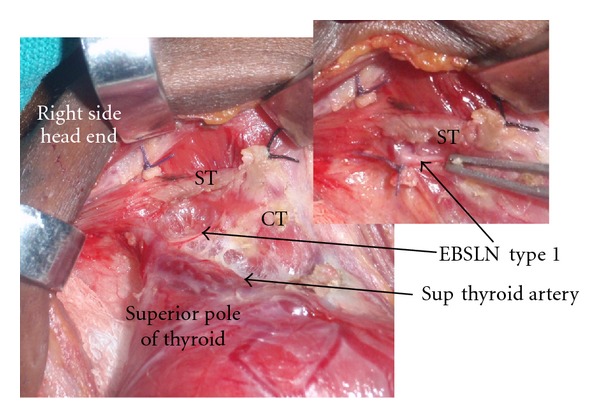
It depicts medial thyroid space with the type 1 EBSLN. Cut upper end of sternothyroid muscle is labelled (ST). CT: cricothyroid muscle, Sup Thy Artery: superior thyroid artery.

EBSLN was described as type 1, IIA, IIB and as “not seen” as per Cernea et al. [4]. EBSLN is dissected in the space medial to the superior pole of the thyroid. The nerve is seen crossing this space and entering the cricothyroid muscle (Figure 1). The branching of the RLN, its relation to inferior thyroid artery (ITA), and the ligament of Berry (LOB) were recorded. The relationship of the RLN to the ITA during its course was recorded as superficial to the artery, deep to the artery, or passing between the branches of the artery. The relationship of the RLN to the LOB was recorded as (A) nerve passing superficial to the ligament, (B) deep to the ligament, (C) through the ligament, and (D) where the nerve splits and passes on either side of the LOB. We dissect the recurrent laryngeal nerve in the trachea-oesophageal groove by the lateral technique or by identifying the nerve at its entry point to the larynx and tracing it to the main trunk of the nerve. The RLN is exposed in all cases and traced. No thyroidectomy is performed without intentional identification and dissection of the laryngeal nerves in our department. Intraoperative neuromonitoring technique was not employed in our study since the authors use it only in recurrent goitres and locally advanced malignancies. The Tubercle of Zuckerkandl was recorded as grade 1 (<0.5 cm or absent), grade 2 (0.5 cm to 1 cm), and grade 3 (>1 cm) [12]. All operations were performed by a single surgeon (PPV).

## 3. Results

**Figure 2:**
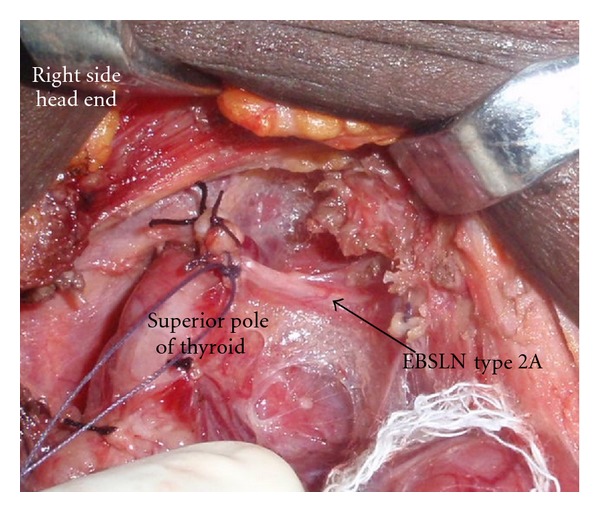
It depicts type 2 EBSLN. Its course is very close to the superior pole of thyroid.

A total of 404 patients who underwent thyroid surgery are included in this study. Among these 180 underwent total thyroidectomy (TT) and 224 patients had hemithyroidectomy (HT). There were 324 nerves on the right side and 260 nerves on the left side in this study. The male to female ratio was 1 : 8. The mean age was 37.52 ± 12.9 years and the mean duration of goitre was 39.49 ± 46.82 months. The indications of surgery included Graves disease, toxic multinodular goitre, early carcinoma thyroid, and non toxic benign goitres. A total of 584 EBSLN and RLN were dissected. Type 1 nerve was the commonest among the 324 right-side EBSLN and 260 EBSLN on the left. The different types of EBSLN are depicted in Table 1. 3.4% of the nerves on the left and 5.4% on the right side were not identified. Figure 1 shows type 1 and Figure 2 show type 2 EBSLN.

**Figure 3:**
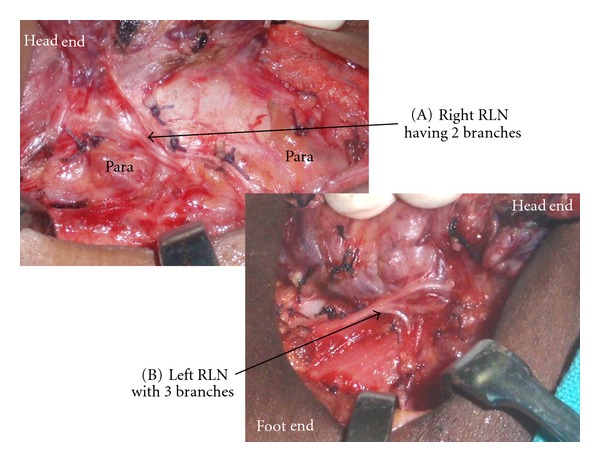
(A) depicts the two branches of right RLN, and (B) shows three branches of left RLN. Para: parathyroid glands.

**Figure 4:**
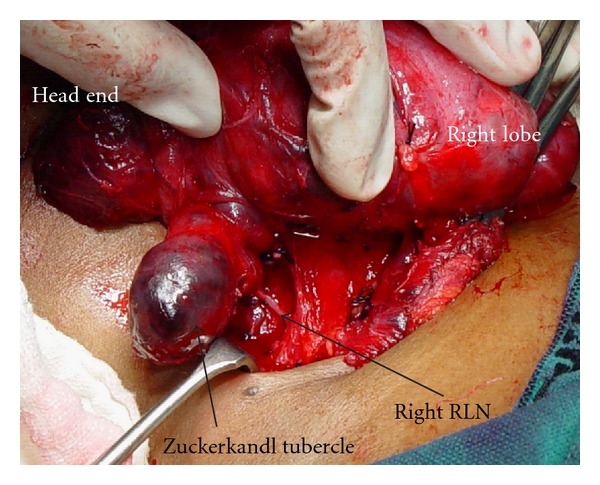
Grade 3 tubercle of Zuckerkandl (TZ). RLN course can be seen coursing medial to the TZ.

RLN continued as a single branch in majority cases: however, around 30% cases had branches. The branching pattern is shown in Table 2.  Figure 3(A) shows two branches and Figure 3(B) shows three branches for the RLN. RLN was deep to ITA in 64.3% (*n* = 375), superficial to ITA in 25.5% (*n* = 161), and between the branches in 8.2% (*n* = 48). The TZ which is an extension from the posterior portion of the thyroid lobes was seen as grade 1 in 65.2% (*n* = 381), grade 2 in 25.1% (*n* = 147), and grade 3 in 9.5% (*n* = 56Figure 4). The RLN was medial to the TZ in 98% of the cases. The relationship of the RLN to the LOB is given in Table 3. In more than 30% of the cases the RLN passed through the LOB. The pyramidal lobe was absent in 21.78% (*n* = 88).

## 4. Discussion

Thyroid surgery was limited to only life-threatening complications arising in the goitre in the early part of the nineteenth century [1, 2] due to the associated mortality. Apart from the introduction of general anaesthesia and antisepsis understanding of the thyroid anatomy and pathology decreased the mortality from thyroid surgeries. As the safety of thyroid surgery increased the complications of the procedure came to limelight and all surgeons concentrated on preventing this. Since the surgical anatomy is now well studied, the morbidity of thyroid surgery has decreased to less than 1%. In this study we describe the nerve course, types, and their variations in 584 laryngeal nerves in our patients undergoing thyroid surgery. Most of the studies on the laryngeal nerve anatomy are cadaveric studies [13]; however, this study demonstrates the anatomy as seen during live surgery in pathologically enlarged glands.

The EBSLN is at risk of injury during thyroid surgeries due to the proximity of the nerve to the superior pole, its variable course and relationship to the superior thyroid artery. It innervates the cricothyroid muscle and tenses the vocal cords. Injury to EBSLN results in the voice fatigue and loss of pitch of voice. This nerve is exposed in the avascular medial thyroid space (Reeves space [1]). Coller and Boyden in 1937 [6] advocated the individual ligation of the superior thyroid vessels and entering the avascular space between the superior pole and cricothyroid muscle {also described by Moosman and De Weese [7]}. Defining this space may be difficult in cases of Hashimotos thyroiditis and advanced carcinoma thyroid. Whitfield et al., in cadaveric dissections have described in detail the site of the entry point of EBSLN into the cricothyroid muscle: however, this is not widely followed for routine EBSLN identification in thyroid surgeries [14]. Similarly detailed description of the relationship between EBSLN, upper part of thyroid gland, and inferior constrictor muscle (IC) of the pharynx was provided to further identify and protect the EBSLN during the thyroidectomy [15]). For descriptive purposes in this study EBSLN is categorized as described by Cernea et al. [4]. According to the Cernea classification type I nerve passes more than 1 cm above the superior thyroid pole, type 2 A crosses <1 cm from the superior pole, and type 2 B at or below the superior pole. Kierner et al. [13] have described yet another classification of nerves as type I to IV. Friedman et al. [8] proposed classification of EBSLN based on its relation to the IC muscle. In our series of cases the incidence of type 1 nerve was higher (Right side 70.7%, left side 72.3%) than the type 2 (25.9% on right versus 23.5% on left). The nerve was not visualised in a few cases (3.4% on the right and 5.4% on the left). This is in contrast to the study on Mishra et al. [16], Aina and Hisham [3], and Ozlugedik et al. [15] who observed higher incidence of type 2 EBSLN in their dissections (63.79%, 82.7%, and 60%, resp.). From our study and the study by Mishra et al. [16], it is evident that there are considerable variations in the EBSLN nerve course even in the same ethnic population group (North versus South Indians). This indicates that intentional nerve identification is a must during thyroidectomy to prevent its injury. The EBSLN can be effectively protected by individual ligation of the branches of the STA. Moosman and De Weese [7] had reported incidence of type 2 nerves in 21% of their cases. It has to be noted that as the gland size increases the incidence of type 2 nerves increases and thereby the chances of injury become high [7]. Estrela et al. [17] demonstrated in cadaveric study that the EBSLN was closer to the cranial point of thyroid lobe and crossed the STA very close to the cranial most part of the thyroid lobe in younger individuals (18–39 years age group). These findings suggest that younger individuals are at higher risk of nerve injury due to the type 2 nature of the EBSLN. Studies done on normal thyroids in the post mortem cases do suggest a different anatomical course for the EBSLN since the anatomy naturally gets distorted when the gland becomes pathologically enlarged [10, 11, 18]. In cadaveric studies it has been shown that the EBSLN gives off many branches above the upper pole of the thyroid gland. The clinically important cricothyroidal branch has been found to be thicker and it pierces the IC muscle about 3.9–17.6 mm above, 3.1–9.9 mm below, or at the level of the upper border of thyroid gland [9]. One or two thyroidal and pharyngeal branches arise from the EBSLN which may be mistaken as cricothyroidal branch; however, these are very thin. Hence, during the routine thyroidectomy the thicker cricothyroidal branch has to be safeguarded [9]. 

Galen in second century A.D described the RLN [19]. Many surgeons including Billroth, Kocher, and Joll tried to avoid this nerve by dissecting away from it; however, some like Bier and Lahey preferred to expose those [1]. Even though the approach to the RLN varied it was realized that the nerve should not be injured. At present the exposure of the nerve is mandatory in all thyroid surgeries. The extra laryngeal branching of the RLN can lead to injuries to some of the branches of the RLN. In our dissection involving 584 RLN's majority had single branch (68.2% on the right side and 70.8% on the left); however, there were two or more branches in 31.42% on the right side and 29.2% on the left. With intraoperative neuromonitoring of the recurrent laryngeal nerve; Serpell et al. [20] had revealed RLN branching in 64.53%. Casella et al. [21] noticed that RLN had branches in 25.7% on the right side and 22.9% on the left. The motor fibers responsible for the adduction and abduction of the vocal cords are located in the anterior branches of the RLN. The RLN branching can be observed before and also after the crossing of the inferior thyroid artery across the nerve. We have observed that the bifurcation of the RLN commonly occurs distal to the crossing with the inferior thyroid artery and hence if the superior approach to identification of RLN is used, one of these branches may be injured. We therefore trace the branches to the trunk of the nerve in cases where we have to use the superior approach to RLN. Branched RLNs represent a risk factor for both temporary and permanent nerve palsy after surgery [22]. The relationship of the RLN to the ITA can vary. In our patients 64.3% is deep to the ITA, 8.2% passes in between the branches of ITA, and the remaining are superficial to the artery (Figure 6(A) and Figure 6(B)). Similarly Berlin [23] observed that more than 80% of the nerves was deep to the ITA.

Tubercle of Zuckerkandl is an important landmark in thyroid surgery. The surgical significance of this tubercle is that the superior parathyroid and RLN can be injured during dissection in the region of TZ. In 34.6% of the cases the tubercle of Zuckerkandl was either grade 2 or 3 in our patients and 98% of these patients had RLN medial to the tubercle. If subtotal thyroidectomy (a common procedure for benign goiters in many of the Indian centres) is performed in patients with grade 2 or 3 TZ it leads to lot of remnant tissue leading on to early recurrence of the goitre. In another series where the identification of TZ was 80%; grade 3 TZ was observed in 45% of cases [12]. They also observed that the RLN was medial to the TZ in 93% cases.

**Figure 5:**
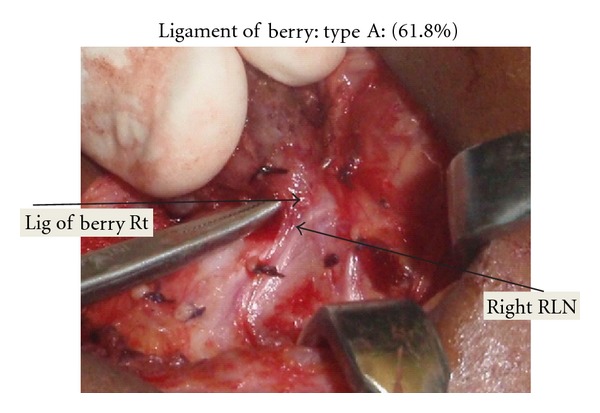
It shows relationship of RLN with ligament of Berry. The nerve is superficial to the LOB in this case.

**Figure 6:**
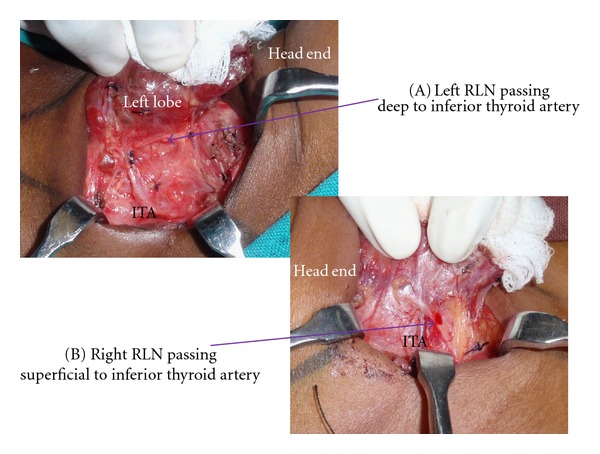
ITA: Inferior thyroid artery. (A) depicts the left RLN deep to the ITA and (B) shows the right RLN superficial to the ITA.

Ligament of Berry anchors the thyroid gland to the laryngotracheal complex [24]. LOB also corresponds to the area where the RLN is most commonly injured [25]. In our series, 31.16% of the RLN passed through the LOB. Even though the dissection of the nerve is not difficult in such cases where the RLN passes through the ligament, the remnant volume to be ablated may be high especially in cases of carcinoma thyroid.  Figure 5 shows the LOB and its closeness to the RLN. It is permissible to leave a very small remnant of tissue in the region of LOB in order to protect the RLN [5].

It is also common to find anatomical variations in such large series of dissections. We observed bilobar thyroid agenesis is one case [25]. Absence of pyramidal lobe, absence of one lobe of the thyroid (*n* = 1), and presence of nonrecurrent laryngeal nerve (*n* = 1) were other variations detected in our series.

The possible limitations of this study are the retrospective nature of the study and no use of IONM. To conclude the modern thyroid surgery is one directed at preventing the morbidity. This is achieved by through knowledge of the surgical anatomy in our patients and taking care of the vital structures like EBSLN, RLN.

## Figures and Tables

**Table 1:** Frequencies of the common types of EBSLN (right side versus left side).

	Right side		Left side	
Type of nerve	Frequency (*n* = 324)	%	Frequency (*n* = 260)	%
Type 1	229	70.7	188	72.3
Type 2A	58	17.9%	41	15.7
Type 2B	26	8	17	6.53
Not identified	11	3.4	14	5.4

**Table 2:** RLN branching pattern (right versus left side).

	Right side		Left side	
No. of branches	Frequency (*n* = 324)	%	Frequency (*n* = 260)	%
Single	221	68.2	184	70.76
Two	99	30.5	71	27.3
Three	03	0.92	05	1.9
Nonrecurrent	1	0.3	Nil	

**Table 3:** Relationship of the RLN to ligament of Berry.

Relationship of the RLN to ligament of Berry	*N* = 584	%
(A) Superficial/away from the ligament	361	61.81%
(B) Deep to the ligament	41	7.02%
(C) Through the ligament	182	31.16%
(D) Splits around the ligament	None	

## References

[1] Bliss RD, Gauger PG, Delbridge LW (2000). Surgeon’s approach to the thyroid gland: surgical anatomy and the importance of technique. *World Journal of Surgery*.

[2] Greene WW (1871). Three cases of bronchocele successfully removed. *American Journal of the Medical Sciences*.

[3] Aina EN, Hisham AN (2001). External Laryngeal Nerve in thyroid surgery: recognition and surgical implications. *ANZ Journal of Surgery*.

[4] Cernea CR, Ferraz AR, Nishio S, Dutra A, Hojaij FC, dos Santos LRM (1992). Surgical anatomy of the external branch of the superior laryngeal nerve. *Head and Neck*.

[5] Delbridge L, Reeve TS, Khadra M, Poole AG (1992). Total thyroidectomy: the technique of capsular dissection. *Australian and New Zealand Journal of Surgery*.

[6] Coller FA, Boyden AM (1937). The development of technique of thyroidectomy: presentation of method used in university hospital. *Surgery, Gynecology & Obstetrics*.

[7] Moosman DA, de Weese MS (1968). The external laryngeal nerve as related to thyroidectomy. *Surgery Gynecology and Obstetrics*.

[8] Friedman M, LoSavio P, Ibrahim H (2002). Superior laryngeal nerve identification and preservation in thyroidectomy. *Archives of Otolaryngology*.

[9] Yalacin B, Tubbs RS, Durmaz A (2012). Branching pattern of the external branch of the superior laryngeal nerve and its clinical importance. *Clinical Anatomy*.

[10] Lennquist S, Cahlin C, Smeds S (1987). The superior laryngeal nerve in thyroid surgery. *Surgery*.

[11] Visset J, Leborgne J, Barbin JY (1975). The external laryngeal nerve. *Bulletin de l’Association des Anatomistes*.

[12] Pelizzo MR, Toniato A, Gemo G (1998). Zuckerkandl’s tuberculum: an arrow pointing to the recurrent laryngeal nerve (constant anatomical landmark). *Journal of the American College of Surgeons*.

[13] Kierner AC, Aigner M, Burian M (1998). The external branch of the superior laryngeal nerve: its topographical anatomy as related to surgery of the neck. *Archives of Otolaryngology *.

[14] Whitfield P, Morton RP, Al-Ali S (2010). Surgical anatomy of the external branch of the superior laryngeal nerve. *ANZ Journal of Surgery*.

[15] Ozlugedik S, Acar HI, Apaydin N, Tekdemir I, Elhan A, Comert A (2007). Surgical anatomy of the external branch of the superior laryngeal nerve. *Clinical Anatomy*.

[16] Mishra A, Temadari H, Singh N, Mishra S, Agarwal A (2007). The external laryngeal nerve in thyroid surgery: rhe “no more neglected” nerve. *Indian Journal of Medical Sciences*.

[17] Estrela F, Leão HZ, Jotz GP (2011). Anatomic relation between the external branch of the superior laryngeal nerve and the thyroid gland. *Brazilian Journal of Otorhinolaryngology*.

[18] Kaplan EL, Salti GI, Roncella M, Fulton N, Kadowaki M (2009). History of the recurrent laryngeal nerve: from Galen to Lahey. *World Journal of Surgery*.

[19] Cernea CR, Hojaij FVC, de Carlucci D (2009). Recurrent laryngeal nerve: a plexus rather than a nerve?. *Archives of Otolaryngology*.

[20] Serpell JW, Yeung MJ, Grodski S (2009). The motor fibers of the recurrent laryngeal nerve are located in the anterior extralaryngeal branch. *Annals of Surgery*.

[21] Casella C, Pata G, Nascimbeni R, Mittempergher F, Salerni B (2009). Does extralaryngeal branching have an impact on the rate of postoperative transient or permanent recurrent laryngeal nerve palsy?. *World Journal of Surgery*.

[22] Lekacos NL, Tzardis PJ, Sfikakis PG, Patoulis SD, Restos SD (1992). Course of the recurrent laryngeal nerve relative to the inferior thyroid artery and the suspensory ligament of Berry. *International Surgery*.

[23] Berlin D (1935). The recurrent laryngeal nerve in total ablation of the normal thyroid gland. *Surgery, Gynecology & Obstetrics*.

[24] Leow CK, Webb AJ (1998). The Lateral Thyroid Ligament of Berry. *International Surgery*.

[25] Kumar TR, Pradeep PV, Ragavan M (2010). Bilobar thyroid agenesis presenting with qdenomatous isthmus and hypothyroidism in a 13 year old girl: a case report. *Journal of Pediatric Sciences*.

